# Occupational Burnout and Insomnia in Relation to Psychological Resilience Among Greek Nurses in the Post-Pandemic Era

**DOI:** 10.3390/bs15020126

**Published:** 2025-01-24

**Authors:** Christos Sikaras, Argyro Pachi, Sofia Alikanioti, Ioannis Ilias, Eleni Paraskevi Sideri, Athanasios Tselebis, Aspasia Panagiotou

**Affiliations:** 1 Nursing Department, Sotiria Thoracic Diseases Hospital of Athens, 11527 Athens, Greece; cris.sikaras@gmail.com; 2 Psychiatric Department, Sotiria Thoracic Diseases Hospital of Athens, 11527 Athens, Greece; olina.alikanioti@gmail.com (S.A.); atselebis@yahoo.gr (A.T.); 3 Department of Endocrinology, Hippocration General Hospital of Athens, 11527 Athens, Greece; iiliasmd@yahoo.com; 4 Nursing Department of General, Hospital of Athens Korgialeneio—Benakeio Hellenic Red Cross, 11526 Athens, Greece; siderhlena@gmail.com; 5 Department of Nursing, University of Peloponnese, 22100 Tripoli, Greece; aspasi65@gmail.com

**Keywords:** occupational burnout, insomnia, psychological resilience, post-COVID era, nurses

## Abstract

Recent studies indicate that nurses experienced high levels of occupational burnout and insomnia during and after the pandemic and highlight resilience as a crucial competence for overcoming adversity. The aim of this study was to assess occupational burnout, insomnia, and psychological resilience and to explore their interrelations among Greek nurses 14 months after the official ending of the pandemic which was declared in May 2023. The study was conducted online in July 2024 and included 380 nurses currently working in Greek hospitals, who completed the Copenhagen Burnout Inventory (CBI), the Athens Insomnia Scale (AIS), and the Brief Resilience Scale (BRS). Overall, 56.1% of the nurses exhibited insomnia symptoms and 46.8% displayed signs of occupational burnout. Low resilience scores were observed in 26.3%. Multiple regression analysis indicated that CBI explained 34.4% of the variance in the AIS scores, while an additional 3% was explained by the BRS. Mediation analysis revealed that resilience operates protectively as a negative mediator in the relationship between burnout and insomnia. In conclusion, one year after the end of the pandemic the levels of occupational burnout and insomnia among nurses remain high, whereas psychological resilience seems to be retained at moderate levels. Consequently, there is an urgent need to regularly monitor for risk of burnout and insomnia and to implement resilience-building strategies for nurses to combat burnout and improve insomnia symptoms.

## 1. Introduction

A multitude of recent studies have highlighted that the COVID-19 pandemic led to unprecedented stress among healthcare professionals (Štěpánek et al., 2023; Tselebis & Pachi, 2022; Sikaras et al., 2023; Hovland et al., 2024; Sasangohar et al., 2020; Baskin & Bartlett, 2021), exacerbating their already burdened mental health (Firew et al., 2020). The long-term psychological impacts remain uncertain (Sikaras et al., 2023). Specifically, contemporary research evidenced that nursing staff were particularly vulnerable to developing physical and psychological issues, experiencing higher levels of occupational burnout and insomnia compared to physicians, possibly because nurses have more prolonged and direct physical interaction with patients (Preti et al., 2020; Jahrami et al., 2022). Thus, this vulnerability might be largely attributed to the unique working conditions of nurses, whose profession involves high and complex physical and psychological demands (Sarafis et al., 2016), such as excessive workload, frequent shift work, and exposure to actual death (Sarafis et al., 2016).

Occupational burnout is described as a workplace phenomenon, which manifests itself as a syndrome linked to emotional and cognitive changes, including emotional exhaustion, depersonalization or cynicism, and reduced personal efficacy due to chronic work-related stress (Arnsten & Shanafelt, 2021). According to Maslach and Jackson, burnout is defined as a response to chronic and long-term workplace stress, characterized by three dimensions: emotional exhaustion, depersonalization, and diminished personal accomplishment (Maslach & Jackson, 1981). Schaufeli and Greenglass further defined it as a state of physical, emotional, and mental exhaustion resulting from prolonged involvement in emotionally demanding work situations (Schaufeli & Greenglass, 2001). Burnout can adversely affect the sleep quality of nursing staff and has been recognized as a cause of insomnia (Parada et al., 2005). Nursing work involves certain characteristics that may increase sleep problems and trigger excessive adrenaline release (Huang et al., 2018; Tselebis et al., 2021). Nurses often work rotating shifts, which can disrupt circadian rhythms (Hsieh et al., 2011) and affect sleep quality (Chang & Peng, 2021). These factors can increase sleep onset latency, prolong the persistence of insomnia, and decrease overall sleep quality and duration (Geiger-Brown et al., 2012; Membrive-Jiménez et al., 2022).

Sleep is essential for life and significantly contributes to health maintenance and well-being. Based on the diagnostic criteria set forth by the 10th Revision of the International Classification of Diseases and Related Health Problems (ICD-10), which most European countries use, insomnia is defined as difficulty in initiating sleep and/or maintaining sleep, or poor sleep quality at least three times a week, for more than a 1-month period. Those who suffer from insomnia worry about the harmful effects of their condition and are highly preoccupied with it, especially at night. Along with a high level of suffering, inadequate sleep duration and quality also interfere with daily functioning. According to the literature, 7–9 h of sleep are recommended to support optimal adult health (Panel et al., 2015). Therefore, it is crucial to examine the relationship between occupational burnout and sleep problems in nurses (Huang et al., 2018). Existing literature points to a reciprocal association between burnout and insomnia, suggesting that either one may be a risk factor for the other (Kwee & Dos Santos, 2023). Also, a recent longitudinal population-based cohort study indicated that burnout was the strongest among several risk factors for insomnia (Höglund et al., 2023).

From a different perspective, psychological resilience may serve as a protective mechanism against the adverse effects of nursing work (Delgado et al., 2017). Due to the nature of their work, nurses encounter challenges that can undermine their resilience (Hart et al., 2012). Resilience is defined as the ability to recover from adversity and overcome difficult life circumstances or, alternatively, as a process of adapting to adversity (McAllister & McKinnon, 2008; Thomas & Revell, 2015). Studies have evidenced that psychological resilience effectively shields healthcare professionals against stress, depression, psychological distress, and burnout (Hart et al., 2012; Pachi et al., 2023b; Lara-Cabrera et al., 2021). Furthermore, recent studies have observed an inverse relationship between resilience and insomnia (Pachi et al., 2024; Palagini et al., 2018). Notably, existing studies indicated that individuals with insomnia showed low resilience (Palagini et al., 2018) and also reported that low resilience levels directly contributed to the severity of insomnia (Cheng et al., 2020). Research conducted during the pandemic demonstrated that healthcare professionals with high resilience levels were better adjusted to recover and withstand psychological strain compared to their less resilient counterparts (Pachi et al., 2023b; Lisi et al., 2020; Labrague, 2021). However, since psychological resilience is a dynamic process, certain factors may weaken it and influence coping strategies (Pachi et al., 2023b; Lisi et al., 2020; Labrague, 2021).

This study was conducted 14 months after the end of the pandemic crisis, which the World Health Organization declared on 5 May 2023 (Pachi et al., 2024). After the official ending of the pandemic several organizational and administrative issues on the healthcare sector in Greece have drastically and unexpectedly aggravated. Firstly, an increased workload was recorded due to a rise in attendance at hospital health services by patients with serious chronic illnesses who had neglected their health because of the pandemic restrictions (WHO, 2020; Huang et al., 2024; Kim et al., 2023; Ghanbari-Jahromi et al., 2024; García-Lara et al., 2024). Subsequently, the temporary healthcare staff departure, since they were recruited to exclusively respond to the needs of the pandemic, resulted in inadequate staffing. These factors in conjunction with the low nurse-to-patient ratio (Mourtou, 2023), the continuous change in nursing policies and health protocols along with the attempts for structural healthcare reforms in the Greek National Health System and the ever-present drawbacks in organizational factors wherein equity and fairness are compromised and safety issues are unresolved, constitute an unfavorable working environment. Likewise, the lack of professional benefits, including salary (Health at a glance, 2023), is a hardship for the nursing workforce resulting in failure to accomplish personal expectations and fulfill occupational goals. Also, after the pandemic period the “quiet quitting” phenomenon of health professionals has increased at an alarming rate and is recently observed among nurses i.e., employees working with less effort, performing only the necessary tasks to avoid being fired, in an attempt to save themselves and maintain a work-life balance (Galanis et al., 2023c). In this context, job burnout emerged as a predictor leading to quiet quitting (Galanis et al., 2023a). Importantly, this gloomy setting, immediately after the pandemic, is placing nurses under constant and chronic workplace stress which is challenging the balance between job demands and job resources. Therefore, this study aimed to assess the levels and investigate the interrelations between occupational burnout, insomnia, and psychological resilience among nurses working in Greek hospitals under regular but stressful and demanding conditions.

Also, a literature review retrieved several studies that have separately examined the effect of burnout and resilience on insomnia. We are not aware of previous research investigating the mediating effect of resilience in the association between burnout and insomnia, thereby leaving a knowledge gap that we have attempted to address. Based on the theoretical framework that enhanced psychological resilience may counteract the harmful effects of burnout on insomnia, we sought to confirm the following research hypotheses:

**Hypothesis** **1:** *Burnout is positively associated with and predicts insomnia*.

**Hypothesis** **2:** *Resilience is negatively related to and predicts insomnia*.

**Hypothesis** **3:** *Resilience serves as a negative mediator in the association between burnout and insomnia*.

## 2. Materials and Methods

### 2.1. Research Design

This was a cross-sectional study involving nurses working in Greek hospitals. Inclusion criteria for the study population were set as a minimum of one year of professional experience. Data collection was performed via self-report questionnaires distributed through email addresses retrieved from scientific and professional registries of Greek nurses. The email invitation contained an anonymous link granting access to the GoogleTM Forms online research platform. Potential participants were fully informed about the purpose of the online survey research. Additionally, we explicitly stated that participation in the survey was voluntary; we ensured the anonymity and confidentiality of responses or other information gathered in the research process emphasizing the implementation of confidentiality measures with secure data storage and access restrictions imposed on the data to protect participant privacy and clarified that prospective participants could voluntarily withdraw from the study at any time, by simply exiting the website and in this instance Google Forms website would have not received their responses. Acceptance of participation, as stated on the first page of the online questionnaire, was considered informed consent. A final approval button should have been checked by the participants to submit their answers to Google Forms and only completed surveys were further processed. Throughout this procedure all relevant limitations and ethical considerations of online surveys were explicitly addressed according to the principles of research ethics (voluntary participation and withdrawal, informed consent, anonymity, confidentiality, autonomy, privacy, risk, and results communication) (Siva Durga Prasad Nayak & Narayan, 2019).

### 2.2. Study Participants

The study was conducted in July 2024. The sample size was computed by Cochran’s formula (included in the Supplementary Materials A). With a target population of 27,103 individuals (Tziallas et al., 2018), a confidence level of 95%, a confidence interval of 5%, and an assumption of a 50% response rate, a minimum sample size of 379 participants was required (Tziallas et al., 2018; Tselebis et al., 2023; Sikaras et al., 2023a; Sikaras et al., 2023b). A total of 500 invitations were emailed, with 380 responses received (response rate: 76%).

The study sample comprised nurses who agreed to participate and responded to the email (convenience sampling). Demographic variables were used to assess the representativeness of the sample as to the broader nursing population in Greece, since a non-probability sampling method was employed (Toepoel & Emerson, 2017). No statistically significant differences were observed between this study sample and the overall population of nurses working in Greece where 83.6% are females (Tziallas et al., 2018), with a mean age of 44.7 ± 9.2 years (Schetaki et al., 2023). Also, since self-report questionnaires were adopted to collect the data, the Harman single-factor test was employed to examine the common method bias (Podsakoff et al., 2003). The most significant component determined 37.438% of the variance which is lower than the criterion of 50%.

### 2.3. Ethical Considerations

The study was conducted following ethical principles outlined in the General Data Protection Regulation (GDPR-2016/679) of the European Union, the World Medical Association Declaration of Helsinki (1975, revised 2008), and the guidelines of the International Committee of Medical Journal Editors. The study protocol was approved by the Ethics Committee of Clinical Research of the General Hospital for Thoracic Diseases of Athens “SOTIRIA” (Approval Number: 20649/23).

### 2.4. Measurement Tools

#### 2.4.1. Demographic Characteristics and Work Experience

Before completing the questionnaires, participants provided demographic and professional information, including gender, age, and years of work experience.

#### 2.4.2. Copenhagen Burnout Inventory (CBI)

The Copenhagen Burnout Inventory (CBI) is a tool designed to measure personal and work-related burnout, consisting of 19 items. Responses include options like “always”, “often”, “sometimes”, “rarely”, and “never/almost never”, or “to a very high degree”, “to a high degree”, “somewhat”, “to a low degree”, and “to a very low degree”. Response options are coded into scores of 100, 75, 50, 0. The possible scoring range for burnout scales is 0–100, with higher scores indicating higher levels of occupational burnout (Kristensen et al., 2005; Sikaras et al., 2021; Pachi et al., 2022). The scale comprises three subscales:

[i]. Personal burnout: Assesses the degree of physical and psychological burnout experienced by the individual (questions 1–6). It refers to both physical and psychological burnout accumulated during the day (e.g., “How often do you feel weak and/or vulnerable to illness”?).

[ii]. Work-related burnout: Evaluates the perceived physical and psychological burnout related to work (questions 7–13). It describes burnout associated with the job (e.g., “Do you feel every hour at work is exhausting”?).

[iii]. Patient-related burnout: Assesses perceived physical and psychological burnout from interaction with patients (questions 14–19). It reflects burnout resulting from interpersonal relationships with patients (e.g., “Do you feel frustrated working with patients”?) (Kristensen et al., 2005; Sikaras et al., 2021; Pachi et al., 2022).

In this study, the Greek version of the CBI, which is a valid scale with good psychometric properties, was used. In the reliability analysis, Cronbach’s alpha exceeded 0.7 for all subscales, indicating a high level of internal consistency (Papaefstathiou et al., 2019). The Cronbach’s alpha coefficient for the entire scale in this study was α = 0.933 and for the Personal burnout subscale the Cronbach’s alpha was 0.903, for the Work-related subscale 0.897, and for the Patient-related subscale 0.849. A total score of ≥50 (Sikaras et al., 2021; Pachi et al., 2022; Henriksen & Lukasse, 2016; Madsen et al., 2015; Hovland et al., 2024; Benson et al., 2009; Chou et al., 2014; Kwan et al., 2021; Creedy et al., 2017) indicates professional burnout. The AVE values of the general scale and the three subscales were 0.5199, 0.529, 0.491 and 0.519, correspondingly The Maslach Burnout Inventory (MBI) and the Copenhagen Burnout Inventory (CBI) have rather different conceptualization of burnout syndrome. The MBI concentrates on three dimensions; namely, emotional exhaustion, depersonalization, and reduced personal accomplishment, whereas the CBI adopts a different strategy by evaluating burnout across the three aforementioned distinct domains. Although the MBI is considered the “gold standard” instrument for measuring burnout, used in numerous studies, it has been challenged for having an ambiguous relationship with the concept of burnout and the designers of the CBI argue that depersonalization and personal accomplishment are not even components of occupational burnout (Kristensen et al., 2005). According to the MBI the emotional aspect of exhaustion represents the core of burnout syndrome, whereas in the CBI the core of burnout is fatigue and exhaustion which shows substantial associations with sleep problems (Kristensen et al., 2005). Another issue is that, in contrast to the MBI, which is regarded as a survey copyrighted or disseminated by a commercial publisher, the CBI is open access and free in the public domain (Alahmari et al., 2022).

#### 2.4.3. Athens Insomnia Scale (AIS)

The Athens Insomnia Scale (AIS) is a self-assessment psychometric tool designed to measure sleep difficulty based on the 10th Revision of the International Classification of Diseases and Related Health Problems (ICD-10). The AIS comprises eight items, with the first five assessing sleep induction (time needed to fall asleep), awakenings during the night, final awakening, total sleep duration, and overall sleep quality. The final three items assess well-being, functioning, and sleepiness during the day. Each item is scored from 0 to 3. A total score of 0 to 24 is possible, with higher scores indicating greater severity of insomnia. A score of 6 or above indicates insomnia (Soldatos et al., 2003). The AIS is a widely used tool for assessing insomnia. The Greek version of the AIS has demonstrated good psychometric properties (Soldatos et al., 2000; Tselebis et al., 2020). Cronbach’s alpha in this study was measured at α = 0.878. The AVE of the scale was 0.544.

#### 2.4.4. Brief Resilience Scale (BRS)

The Brief Resilience Scale (BRS) is a tool designed to measure resilience. It comprises six items (three positive and three negative), with responses on a 5-point Likert scale (ranging from “strongly disagree” to “strongly agree”). The positive questions assess the ability to recover from adverse situations (e.g., “I tend to bounce back quickly after hard times”), while the negative questions assess difficulties in overcoming adverse events (e.g., “It is hard for me to snap back when something bad happens”). The total BRS score is calculated by summing the scores of the six items and dividing by the number of responses. Possible scores range from 1 to 5, with higher scores indicating greater resilience (Smith et al., 2008; Kyriazos et al., 2018). Low scores range from 1.00 to 2.99, medium scores from 3.00 to 4.30, and high scores from 4.31 to 5.00 (Kyriazos et al., 2018). The BRS has shown high reliability and validity and is frequently used in studies with healthcare professionals (Tselebis et al., 2020). The Greek version of the BRS has been validated, demonstrating high reliability and validity in measuring resilience (Kyriazos et al., 2018). Cronbach’s alpha in this study was measured at α = 0.875. The AVE of the scale was 0.617.

### 2.5. Statistical Analysis

Descriptive statistical methods were employed to estimate means and standard deviations for continuous variables. Subsequently, we examined the representativeness of the sample using *t*-test and χ^2^ test, comparing the sample with the general population of Greek hospital nurses in terms of age, and gender. Then, common method bias testing was necessary, using the Harman single factor method test, because the study’s data were derived from self-reports. Further, we assessed the presence of gender differences as to the study variables using *t*-tests. Correlations were investigated using Pearson’s correlation test. Linear regression analysis was utilized to determine if the correlated variables were significant predictors of insomnia. Before proceeding with the regression, linearity was confirmed through visual inspection of scatter plot pairs, normality was checked using P-P plots, and homoscedasticity was verified through a residual scatterplot. The Durbin-Watson test was employed to assess the independence of residuals, while the absence of multicollinearity in the data was examined using Variance Inflation Factor (VIF) analysis. Mediation analysis was conducted using Hayes’ SPSS Process Macro Model 4. In the mediation analysis, the outcome variable was AIS, the mediator variable was BRS, and the predictor variable was CBI (total). All statistical analyses were performed using IBM SPSS Statistics 21 (IBM SPSS Statistics for Windows, Version 21.0, Armonk, NY, USA: IBM Corp.). Mediation analysis was conducted using the Hayes SPSS Process Macro version 4.0. Statistical significance was set at *p* < 0.05 (two-tailed). IBM SPSS AMOS 21 Graphics enabled the graphical representation of the confirmatory factor analysis of the CBI and the path model of the study variables, with all the data from the fit model (included in the Supplementary Materials B and C).

## 3. Results

### 3.1. General Characteristics of Participants and Scores on Outcome Variables as to Gender

The study included 74 male and 306 female nurses. In the sample, 56.1% showed symptoms of insomnia (AIS ≥ 6), while 46.8% exhibited signs of burnout (CBI Total ≥ 50). Low resilience scores were seen in 26.3% of the sample (BRS ≤ 2.99), while high scores were observed in 16.6% (BRS ≥ 4.31). Table 1 presents the mean values and standard deviations of the study variables.

Regarding gender, female nurses had a higher mean burnout score compared to male nurses (*t*-test *p* < 0.05, 49.64 ± 19.03 vs. 44.91 ± 17.39, Table 1). Additionally, female nurses showed higher scores in both personal burnout and work-related burnout (Table 1).

### 3.2. Correlations Among Continuous Variables

AIS correlated negatively with the BRS and positively with the CBI total, as well as with its three subscales, confirming the associations of the first and second hypothesis. BRS showed a negative correlation with both the CBI total and its subscales. As expected, the three CBI subscales positively correlated with each other (Pearson Correlations *p* < 0.01, Table 2). Work experience exhibited weak positive association with the BRS and weak negative correlation with the AIS.

### 3.3. Multiple Regression Analysis

Before proceeding to regression analysis, we checked if the necessary assumptions for regression analysis were met. Independence of residuals was tested using the Durbin-Watson test, with a value of 1.92 (Table 3), supporting the absence of autocorrelation. The VIF value of 1.264, less than 4, indicated a lack of multicollinearity (Table 3). Normality was verified by visual inspection of the predicted probability plots. Homoscedasticity was examined through visual inspection of the scatter plot of standardized and predicted residual values. Linearity was confirmed by visually inspecting scatter plots of variable pairs.

We conducted a multiple regression analysis using the Stepwise method to explore which factors best explain the scores of the Athens Insomnia Scale (AIS). In the multiple regression, AIS was set as the dependent variable, while age, gender, years of work experience, the Copenhagen Burnout Inventory (CBI Total), and the Brief Resilience Scale (BRS) were set as independent variables. The analysis showed that CBI Total explained 34.4% of the variance in AIS, while an additional 3% was explained by BRS (Table 3), verifying the first and second hypothesis. The remaining variables (age, gender and years of work experience) did not play a statistically significant role in explaining AIS.

### 3.4. Simple Mediation Analysis

**Figure 1:**
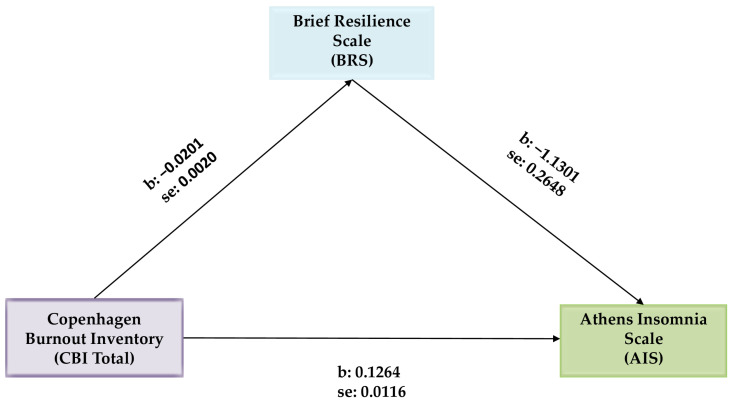
Mediation analysis of the Brief Resilience Scale (BRS) on the Copenhagen burnout inventory (CBI Total) and the Athens Insomnia Scale (AIS) relationship.

Next, we explored the hypothesis that BRS might act as a mediator in the relationship between CBI Total and AIS. In this analysis, CBI Total was set as the predictor variable, BRS as the mediator variable, and AIS as the outcome variable. Covariates included work experience and age. Hayes’ SPSS Process Macro Model 4 was used, with the analysis based on 5000 bootstrap samples. Unstandardized coefficients for the variables with standard errors are illustrated in Figure 1.

The mediation analysis revealed that BRS acts as a mediator in the relationship between CBI Total and AIS, thus confirming the third hypothesis. In this context, the covariates, age, and work experience, did not exhibit statistically significant relationships (Figure 1 and Table 4). The indirect effect of BRS was statistically significant [b = 0.0227, 95% CI (0.0112, 0.0362), *p* ≤ 0.01]. Furthermore, even in the presence of the BRS mediator, the direct effect of CBI total on AIS remained significant [b = 0.1264, 95% CI (0.1035, 0.1493) *p* ≤ 0.001)]. This model explains 15% of the variance in the AIS outcome variable.

## 4. Discussion

### 4.1. Summary of Results Compared with Other Studies

This study was conducted 14 months after the World Health Organization officially declared the end of the COVID-19 pandemic. In this context, in the presence of significant factors affecting the quality and safety of nursing care and the well-being of nurses, this descriptive correlational study depicts the current situation of nursing staff by assessing the levels of occupational burnout, insomnia, and psychological resilience among Greek hospital nurses. The findings of the present study indicate that both burnout and insomnia levels among nurses remain high. Regarding occupational burnout, results from this research are similar to those of a study conducted one year after the onset of the pandemic (February 2021) involving nurses in Greek hospitals (*t*-test *p* > 0.05, 48.72 ± 18.89 vs. 46.95 ± 18.75) (Sikaras et al., 2021), as well as to another study conducted around the same period (the second half of March 2021) in Greek hospitals (Pachi et al., 2022). In terms of insomnia, this study recorded significantly higher levels compared to a study conducted two months after the onset of the pandemic (May 2020) among nurses in Greek hospitals (*t*-test *p* < 0.001, 7.12 ± 4.24 vs. 5.98 ± 4.24, Hedges’ g: 0.27) (Tselebis et al., 2020). These findings are also comparable to those of a study conducted in approximately two years after the onset of the pandemic among Greek hospital nurses (Sikaras et al., 2023a), and another study conducted two months after the pandemic ended (Pachi et al., 2024).

Concerning psychological resilience, this study evidenced significantly lower levels compared to a study conducted during the first wave of the pandemic (June 2020) involving nurses and doctors in Greece (*t*-test *p* < 0.01, 3.43 ± 0.83 vs. 3.61 ± 0.8, Hedges’ g: 0.22) (Pachi et al., 2023a). According to another study carried out two months after the end of the pandemic among Greek hospital nurses (Pachi et al., 2024), resilience levels were similar to those in the present study (mean ± SD = 3.39 ± 0.78). Comparable results (mean ± SD = 3.50 ± 0.7) were also found in the study by Moisoglou et al., conducted two and a half years after the onset of the pandemic among Greek hospital nurses (Moisoglou et al., 2024b).

The persistence of high burnout and insomnia may be attributed, among other factors, to the reduced perceived organizational support for nurses, while the impact of the pandemic still seems to linger (Moisoglou et al., 2024b; Abdulmohdi, 2023). Moreover, the severe shortage of nursing staff in the Greek National Health System (Health at a glance, 2020), coupled with challenging working conditions, and the low wages of Greek nurses compared to their counterparts in other OECD countries (Health at a glance, 2023), are significant contributors to the situation. Additionally, the increased perceived family support experienced by the Greek nurses during the first wave of the pandemic when nurses were celebrated as “heroes”, seems to have diminished after one year, as evidenced in results from studies conducted in June 2020 and in November–December 2021, which may have undermined the protective role of family support on nurses’ mental health (Sikaras et al., 2023a; Bratis et al., 2009; Pachi et al., 2023a). Furthermore, a recent study indicated that Greek nurses exhibited higher levels of burnout compared to other healthcare professionals i.e., physicians, midwives, psychologists, pharmacists, etc., even in the post-pandemic era (Galanis et al., 2023b).

Likewise, the moderate levels of resilience observed in this study may be due to the perceived lack of organizational support (Moisoglou et al., 2024b; Abdulmohdi, 2023), the severe shortage of nursing staff and the challenging working conditions (Health at a glance, 2020), the low salaries of nurses in Greece (Health at a glance, 2023), and possibly because the increased perceived family support recorded during the first wave of the pandemic among Greek nurses, is no longer being experienced (Labrague, 2021; Hovland et al., 2024). According to a review article by Baskin and Bartlett, the psychological resilience of healthcare professionals worldwide during the pandemic was found to be at moderate levels (Baskin & Bartlett, 2021). Data analysis revealed a decline in resilience among U.S. nurses during the pandemic, while Chinese nurses showed increased resilience compared to pre-pandemic levels (Baskin & Bartlett, 2021). As indicated by Jo et al., in a cross-sectional study based on international data, organizational support and nurse participation in policy development during the pandemic enhanced their resilience (Jo et al., 2021). The same study found that U.S. nurses exhibited higher resilience compared to their counterparts in Japan, Turkey, and Korea (Jo et al., 2021).

### 4.2. Levels of Occupational Burnout as to Gender

Regarding gender, no statistically significant differences were found in insomnia and psychological resilience. However, female nurses displayed higher levels of occupational burnout overall and specifically in the dimensions related to personal and work-related burnout, consistent with findings from previous studies (Sikaras et al., 2021; Pachi et al., 2022). Female gender is presumed to be one of the leading factors that might influence the recorded impact of burnout among nurses (Woo et al., 2020). Existing studies and meta-analysis report higher levels of burnout among female nurses compared to males (Batra et al., 2020; Alenezi et al., 2024), but results remain inconclusive since other research studies and meta-analysis evidenced contradictory findings on gender differences as to burnout and stated that male nurses are more prone to experiencing burnout compared to females (la Fuente et al., 2018; Zhang et al., 2022). Among other factors, it is possible that the use of different validated tools to measure burnout has highlighted different aspects of the syndrome and has led to differences in its conceptualization and recorded expression (la Fuente et al., 2015).

### 4.3. The Effect of Occupational Burnout on Insomnia

This study showed significant positive correlations between occupational burnout (particularly the subscales related to personal and work-related burnout) and insomnia. These findings align with the literature that supports this relationship (Wolkow et al., 2019; Söderström et al., 2012; Armon et al., 2008; Weaver et al., 2020). Furthermore, multiple regression analysis in this study indicated that occupational burnout could explain 34.4% of the variance in insomnia. This finding is consistent with research suggesting that burnout can affect the quality of sleep and is recognized as a cause of insomnia (Membrive-Jiménez et al., 2022; Salvagioni et al., 2017). A prospective study evidenced that burnout at baseline not only exacerbated insomnia symptoms over time for individuals already exhibiting these symptoms at baseline, but it was also linked to the emergence of new insomnia cases at follow-up (Armon et al., 2008). Possible ways that burnout may contribute to insomnia include heightened pre-sleep arousal, intrusive thoughts and worries that prevent relaxation, and psychosomatic symptoms such as headaches and digestive symptoms that can interfere with sleep (Kuriyama, 2022; Sørengaard & Saksvik-Lehouillier, 2022; Zarei & Fooladvand, 2022; Khammissa et al., 2022). In contrast, sleep deprivation alone cannot cause burnout but can precipitate and/or exacerbate it (Zarei & Fooladvand, 2022; Metlaine et al., 2017). Insomnia and burnout have a bidirectional relationship, similar to other mental health conditions. Reduced sleep duration and/or poor sleep quality can affect burnout, while burnout can also impact on sleep duration and quality (Wolkow et al., 2019; Söderström et al., 2012; Armon et al., 2008; Weaver et al., 2020). Sufficient and good-quality sleep can help address occupational burnout (Assefa et al., 2015). As stated, chronic depletion of individual energy reserves, influenced by the continuous activation of the hypothalamic-pituitary-adrenal axis and increased stress levels, can lead to both sleep disturbances and burnout (Stewart & Arora, 2019).

### 4.4. The Influence of Psychological Resilience on Insomnia

Regarding psychological resilience, this study found that it was negatively associated with both occupational burnout and insomnia, highlighting its positive influence. Additionally, multiple regression analysis showed that resilience could explain 3% of the variance in insomnia. These findings align with results from other studies supporting the protective role of resilience against burnout and insomnia in nurses (Pachi et al., 2024; Moisoglou et al., 2024b), as well as in the general population’s mental health (Ye et al., 2020b; Li et al., 2021). A recent study highlighted the significant beneficial effect of resilience in patients with post-COVID-19 syndrome, who were found to experience better mental health and quality of life (Moisoglou et al., 2024a).

Specifically for insomnia, research supports that resilience is closely linked to sleep quality (Arora et al., 2022) and greater resilience is associated with higher sleep efficiency as objectively recorded via sleep electroencephalograms (Brand et al., 2014). Also, another research study reported that resilience is a protective factor for sleep quality (Wang et al., 2020). An earlier study considering sleep disturbance as the dependent variable and resilience as the predictor variable, suggested that when perceived stress was present, a high level of resilience protected against sleep disturbance (Liu et al., 2015). Moreover, a recent longitudinal study evidenced that sleep quality during the second wave of the pandemic was predicted by resilience levels measured during the first wave (Lenzo et al., 2022). Furthermore, a recent cross-sectional study identified resilience as a predictor of sleep quality among newly employed nurse (Lin et al., 2023). A plausible explanation of how resilience influences insomnia implicates the cognitive process of rumination. A recent study supported that the association between rumination and sleep quality is moderated by resilience (Li et al., 2019). Accordingly, the impact of stressful life events and rumination on sleep quality is expected to decrease at high resilience levels and increase at low levels of resilience (Li et al., 2019).

### 4.5. The Mediating Role of Psychological Resilience

Furthermore, mediation analysis evidenced that resilience operates as a negative mediator in the relationship between burnout and insomnia. A recent meta-analysis indicated that resilience and burnout are inversely correlated (Castillo-González et al., 2023). A recent review showed that resilience effectively counteracted the effects of burnout among critical care nurses (Olaleye et al., 2022). Studies reported that resilient healthcare workers have less burnout (Ferreira & Gomes, 2021; Di Giuseppe et al., 2021; Mao et al., 2024) and the results from a cross-sectional research evidences that an increase in nurses’ resilience contributed to mitigating signs of burnout (Jose et al., 2020). As stated in a research study, resilience reduced the experience of fatigue and effectively buffered, up to a certain threshold, the negative consequences of rumination amongst college students during the COVID-19 pandemic (Ye et al., 2020a), whereas other authors argued that resilience moderated the positive association between rumination and perceived stress and in this way, indirectly, effectuated sleep improvements (Du et al., 2020). Undoubtedly, less rumination at night will result in better sleep quality (Tousignant et al., 2018) and this explanation offers a possible speculation about the protective role of resilience.

### 4.6. Theoretical and Practical Implications

This descriptive correlational study provided information on the prevalence of burnout, insomnia and resilience among Greek nurses, 14 months after the pandemic conclusion, which suggests that hospital managers should implement interventions to decrease burnout and insomnia symptoms and foster resilience. Also, the results revealed possible pathways which may indicate potential management strategies, since they contribute to understanding the interrelations between resilience, burnout and insomnia and confirm the vital role of resilience successfully counteracting the negative consequences of burnout on insomnia. Several strategies fostering resilience effectuate significant improvements in managing burnout, such as mindfulness practices to increase awareness of workplace stressors and emotion regulation through self-compassion techniques, training in positive coping and development of problem-solving and communication skills (Mendy, 2020; Magtibay et al., 2017; Kayalar & Hiçdurmaz, 2024; Şenocak & Demirkıran, 2023; Hardie et al., 2021). Also, stress management programs based on psychoeducation could improve sleep and mitigate burnout (Hittle et al., 2023; Ekstedt et al., 2009; Sawyer et al., 2023; Sharin et al., 2024), as could self-care programs (Morimoto et al., 2015; Kravits et al., 2009).

Enhancing resilience has a multifaceted positive influence on nurses, protecting them not only from burnout but also from other negative aspects of their profession (Kılınç & Çelik, 2020; Peng et al., 2021). During the pandemic, the shielding effect of resilience against burnout, especially when nurses did not receive adequate support from their supervisors, was significant (Phillips et al., 2022). Strengthening resilience is a necessary and important intervention effectuating improvements on nurses’ well-being and can be cultivated (Foster et al., 2018; Foureur et al., 2013). Resilience is a dynamic process that evolves over time and involves an adaptive functioning style that allows individuals to cope with difficulties. It may start with recognizing that negative emotions such as sadness, anger, or anxiety are natural consequences of being human in the midst of a crisis and not necessarily problems that simply need to be amended (Sisto et al., 2019; Barthélemy et al., 2020).

The unresolved workplace stressors, the reduced teamwork dynamics, role conflict, job instability, the aging population of the nursing workforce, the nursing shortage and the increasing demands of patients, the political attitudes regarding healthcare and the lack of sufficient financing to provide appropriate services, along with workplace violence and bullying (Galanis et al., 2024), especially towards the less experienced nurses (Lin et al., 2023; Baek & Lee, 2022), are factors adding to workplace adversity and contributing to the increased levels of burnout. Research comparing burnout levels before and during the pandemic stated that under normal pre-pandemic working conditions nurses had significant rates of burnout, but, during the pandemic, even higher rates of burnout were being documented (Sullivan et al., 2021). Similarly, studies recording rates of burnout during and after the pandemic are required to disentangle the pandemic effect.

Therefore, nurse managers and health policymakers should be aware of these problems to implement timely and preventive interventions to improve working conditions (Galanis et al., 2024; Jackson et al., 2018). It is crucial that organizations recognize the magnitude of the issue, as addressing burnout caused by organizational factors will likely be more effective than isolated individual interventions (De Simone et al., 2019). The organizational support for nurses (Abdulmohdi, 2023) finds practical application in the Collective Resilience Intervention Model which was developed to emphasize the role of organizational structural procedures in fostering resilience (Rees et al., 2015; Heritage et al., 2019). Thus, there is an urgent need to improve support for nurses from their leaders and employers, including recognition of their work, moral and financial support, ensuring adequate staffing to reduce workload and shift work, and applying meritocracy and justice in their management. Finally, nursing authorities and leaders should propose and introduce programs for the timely and accurately detection of burnout and insomnia symptoms and for the regular assessment and enhancement of nurses’ resilience to protect them from burnout and sleep disorders (Alameddine et al., 2021).

Despite its strong theoretical and methodological basis, this study has certain limitations. Firstly, the cross-sectional nature of this study capturing information at one point in the time only, did not allow us to establish causal relationships between occupational burnout, insomnia, and psychological resilience, requiring caution in interpreting the results. Longitudinal studies are needed to more accurately determine the overtime changes of the levels and the relationships among the aforementioned variables. Only with prospective studies measuring these variables at different times throughout the pandemic and after its conclusion we would be able to separate the effect of the pandemic from that of other common stressors on healthcare practices. Secondly, data collection was conducted through self-reported questionnaires, which, while valid, may still carry the potential for response bias. Thus, nurses who suffered from burnout and/or insomnia could be more or less motivated to participate in the survey and could also be subject to recall bias which could have affected the retrospective self-reports. Likewise, it is impossible to fully eliminate social desirability bias in online self-reported questionnaires, although we assured the anonymity and confidentiality. To mitigate these bias objective metrics could be an asset to future research. Thirdly, the absence of clinical interviews or objective data to confirm subjective differences, and the use of convenience sampling, necessitate caution in interpreting or generalizing the results. Although we attempted to compare our sample to the total population of Greek hospital nurses, several sociodemographic variables were missing, which compromises the degree of confidence in the representativeness of our findings. Also, the study sample was derived only from Greek hospital nurses, which may limit the generalization of the findings to other countries with different operational environments and cultures. Moreover, an important drawback was the absence of information on the nurses’ work department, work shifts, rotation and staff shortages. Finally, this study examined specific risk and protective factors affecting insomnia, namely burnout and resilience, with limited covariates. To further understand their intricate relationships beyond the pandemic, future studies may include more risk (other stress-related dimensions) and protective factors (family or social support) (Tselebis et al., 2013; Jarrin et al., 2014) to guide the design of tailored interventions and address the diverse aspects of nurses’ well-being.

## 5. Conclusions

As we move on to the second year after the end of the pandemic, with its impact on health systems diminished, it seems that other factors have emerged and are exerting a negative influence on nurses. Results from this study evidence that both burnout and insomnia levels among nurses remain high, since more than half of nurses suffer from insomnia symptoms and almost half of them exhibit signs of burnout. Psychological resilience among nurses also appears to remain at moderate levels with more than one in four nurses reporting low resilience. Regression analysis determined that burnout accounted for 34.4% of the variance in the insomnia, whereas resilience contributed an additional 3%. Mediation analysis confirmed that resilience operates as a protective negative mediator in the relationship between burnout and insomnia. The above findings underscore the urgent need to address occupational burnout and insomnia among nurses and simultaneously strengthen their psychological resilience. Additionally, it is crucial for nursing authorities and leaders to propose and introduce programs for the regular assessment for risk of burnout and insomnia among nursing staff and implement strategies to consistently enhance nurses’ resilience.

## Figures and Tables

**Table 1:** Descriptive statistics of participants.

Gender		Age	Work Experience (in Years)	Athens Insomnia Scale	Brief Resilience Scale	Copenhagen Burnout Inventory			
						Total	Personal Burnout	Work Related Burnout	Patient Related Burnout
Male	Mean	47.57 *	21.89	6.35	3.58	44.91 *	44.76 **	47.83 **	41.67
	N	74	74	74	74	74	74	74	74
	S.D.	10.85	11.92	4.23	0.89	17.93	18.71	20.89	21.26
Female	Mean	44.58 *	19.92	7.31	3.40	49.64 *	52.67 **	55.45 **	39.84
	N	306	306	306	306	306	306	306	306
	S.D.	10.41	11.47	4.92	0.81	19.03	19.86	22.74	23.68
Total	Mean	45.16	20.30	7.12	3.43	48.72	51.13	53.97	40.20
	N	380	380	380	380	380	380	380	380
	S. D.	10.55	11.57	4.80	0.83	18.89	19.87	22.56	23.21

* *t* test *p* < 0.05, ** *t* test *p* < 0.01.

**Table 2:** Correlations among age, work experience, AIS, BRS and CBI.

Pearson Correlation N: 380		Age	Work Experience (in Years)	AIS	BRS	CBI Total	CPB	CWB	CPRB
Work experience (in years)	r	0.894 **							
	*p*	0.001							
Athens Insomnia Scale (AIS)	r	−0.064	−0.126 *	0.7375					
	*p*	0.214	0.014						
Brief Resilience Scale (BRS)	r	0.080	0.111 *	−0.423 **	0.7855				
	*p*	0.120	0.030	0.001					
Copenhagen Burnout Inventory (CBI Total)	r	−0.031	−0.058	0.587 **	−0.457 **	0.7210			
	*p*	0.552	0.257	0.001	0.001				
Copenhagen Personal Burnout (CPB)	r	−0.024	−0.068	0.634 **	−0.449 **	0.866 **	0.7373		
	*p*	0.642	0.183	0.001	0.001	0.001			
Copenhagen Work-related Burnout (CWB)	r	−0.029	−0.047	0.567 **	−0.397 **	0.919 **	0.789 **	0.7007	
	*p*	0.578	0.362	0.001	0.001	0.001	0.001		
Copenhagen Patient-related Burnout (CPRB)	r	−0.026	−0.039	0.327 **	−0.343 **	0.794 **	0.482 **	0.559 **	0.7204
	*p*	0.615	0.454	0.001	0.001	0.001	0.001	0.001	
AVE (Average Varience Extacted)				0.544	0.617	0.5199	0.529	0.491	0.519
CR (Composite Reliability)				0.904	0.906	0.9532	0.917	0.743	0.865

* Pearson Correlations *p* < 0.05, ** Pearson Correlations *p* < 0.01. Note: The square roots of the AVE are placed on the diagonal.

**Table 3:** Stepwise multiple regression.

Dependent Variable: Athens Insomnia Scale	*R* Square	*R* Square Change	*Beta*	*t*	*p*	VIF	Durbin-Watson
Copenhagen Burnout Inventory (CBI Total)	0.344	0.344	0.497	10.863	0.001 *	1.264	1.918
Brief Resilience Scale	0.375	0.030	−0.195	−4.267	0.001 *	1.264	

Notes: *Beta* = standardized regression coefficient; * Correlations are statistically significant at the *p* < 0.001 level (only statistically significant variables are included).

**Table 4:** Mediation analysis ** of the Brief Resilience Scale (BRS) on the Copenhagen Burnout Inventory (CBI Total) and the Athens Insomnia Scale (AIS) relationship *.

*Variables*	*b*	*SE*	*t*	*p*	*95% Confidence Interval*	
					LLCI	ULCI
CBI Total → BRS	−0.0201	0.0020	−9.9928	0.001	−0.0240	−0.0161
CBI Total → AIS	0.1264	0.0116	10.8629	0.001	0.1035	0.1493
CBI Total → BRS → AIS	−1.1301	0.2648	−4.2673	0.001	−1.6509	−0.6094
*Effects*						
Direct	0.1264	0.0116	10.8629	0.001	0.1035	0.1493
Indirect *	0.0227	0.0064			0.0113	0.0362
Total	0.1491	0.0106	14.0899	0.001	0.1283	0.1699

Notes: * based on 5000 bootstrap samples. ** Included in the Supplementary Materials D.

## Data Availability

The data that support the findings of this study are available from the corresponding author, [A.T.], upon reasonable request.

## Supplementary Materials

The following supporting information can be downloaded at: https://www.mdpi.com/article/10.3390/bs15020126/s1.
