# Supplementary material for: Occupational Burnout and Insomnia in Relation to Psychological Resilience Among Greek Nurses in the Post-Pandemic Era
Source: Behav Sci (Basel). 2025 Jan 24;15(2):126. doi: 10.3390/bs15020126 (PMC11851848; doi:10.3390/bs15020126)
Supplement: Supplementary file 1 [file behavsci-15-00126-s001.zip › behavsci-3325324-supplementary.pdf]

Supplementary Materials

A. Formula for determining sample size

$$\text{Sample size, n} = \text{N} * \frac{\frac{Z^2 * p * (1-p)}{e^2}}{[N - 1 + \frac{Z^2 * p * (1-p)}{e^2}]}$$

z is the z score= 1.96
e is the margin of error=0.05
N is the population size=27103
p is the population proportion=0.5

Confidence Level	z-score (±)
0.92	1.75
0.95	1.96

B. Path Analysis

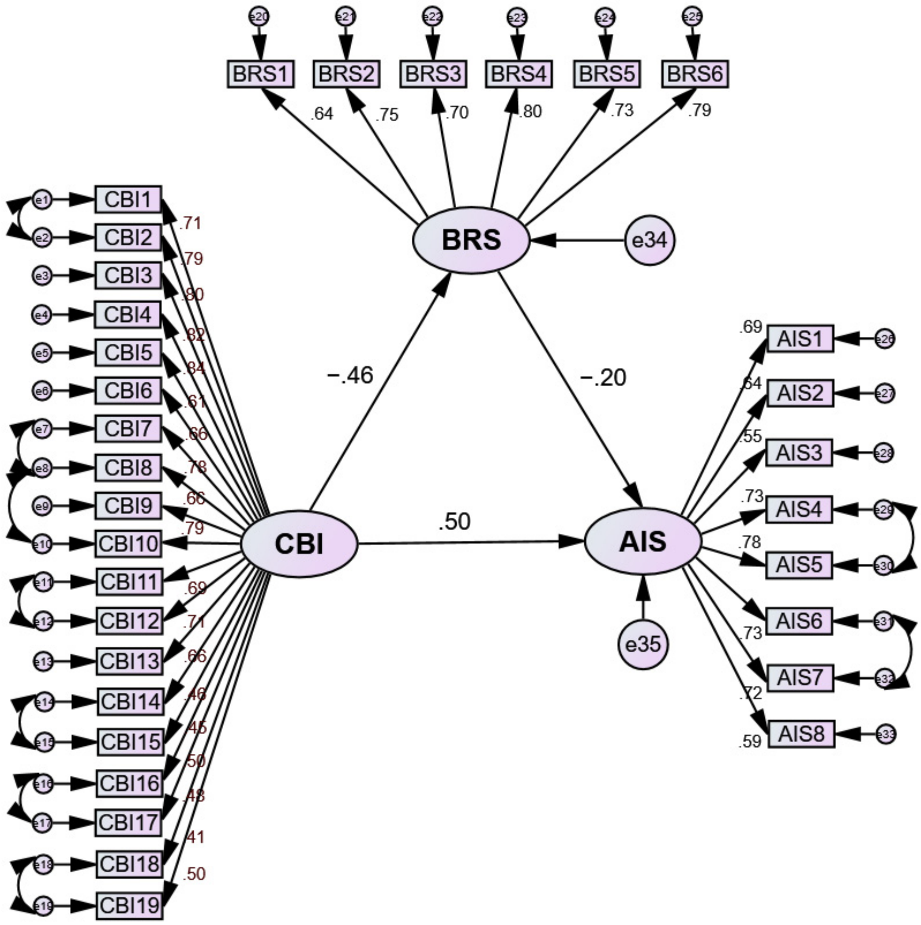

B. Model Fit Summary

Standardized RMR = 0.0538

CMIN

Model	NPAR	CMIN	DF	P	CMIN/DF
Default model	91	1009.962	470	.000	2.149
Saturated model	561	.000	0		
Independence model	33	8107.697	528	.000	15.355

RMR, GFI

Model	RMR	GFI	AGFI	PGFI
Default model	30.912	.860	.832	.720
Saturated model	.000	1.000		
Independence model	193.182	.185	.134	.174

Baseline Comparisons

Model	NFI Delta1	RFI rho1	IFI Delta2	TLI rho2	CFI
Default model	.875	.860	.929	.920	.929
Saturated model	1.000		1.000		1.000
Independence model	.000	.000	.000	.000	.000

Parsimony-Adjusted Measures

Model	PRATIO	PNFI	PCFI
Default model	.890	.779	.827
Saturated model	.000	.000	.000
Independence model	1.000	.000	.000

NCP

Model	NCP	LO 90	HI 90
Default model	539.962	452.217	635.442
Saturated model	.000	.000	.000
Independence model	7579.697	7291.566	7874.250

FMIN

Model	FMIN	F0	LO 90	HI 90
Default model	2.665	1.425	1.193	1.677
Saturated model	.000	.000	.000	.000
Independence model	21.392	19.999	19.239	20.776

RMSEA

Model	RMSEA	LO 90	HI 90	PCLOSE
Default model	.055	.050	.060	.038
Independence model	.195	.191	.198	.000

AIC

Model	AIC	BCC	BIC	CAIC
Default model	1191.962	1209.898	1550.518	1641.518
Saturated model	1122.000	1232.574	3332.436	3893.436
Independence model	8173.697	8180.201	8303.723	8336.723

ECVI

Model	ECVI	LO 90	HI 90	MECVI
Default model	3.145	2.914	3.397	3.192
Saturated model	2.960	2.960	2.960	3.252
Independence model	21.566	20.806	22.344	21.584

HOELTER

Model	HOELTER .05	HOELTER .01
Default model	196	205
Independence model	28	29

C. Confirmatory Factor Analysis of the CBI

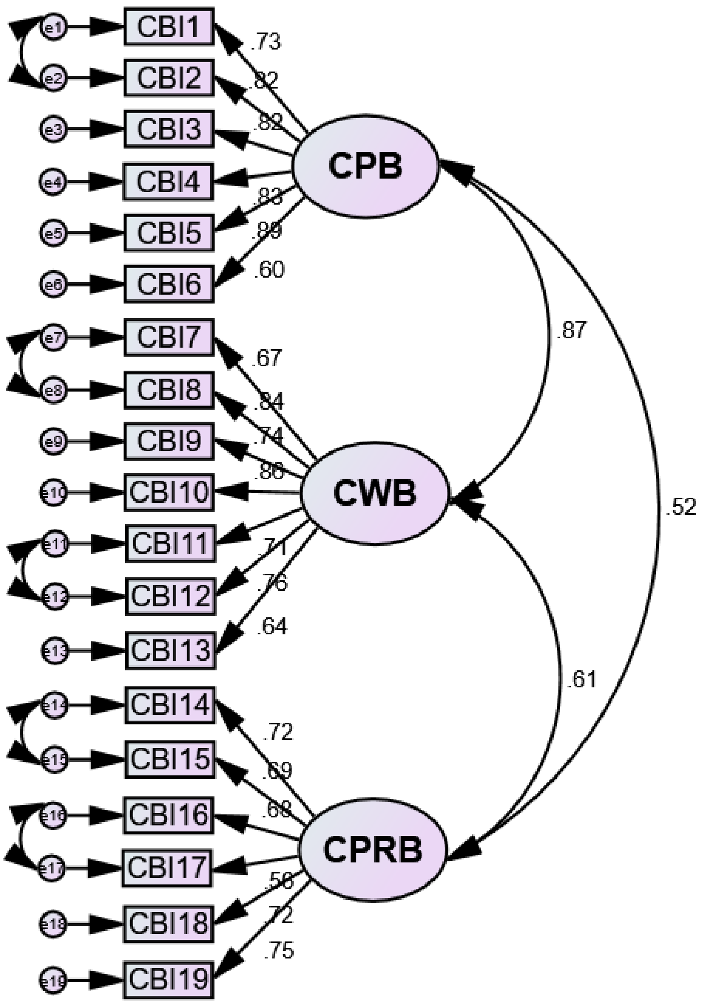

C. Model Fit Summary

Standardized RMR = 0.0544

CMIN

Model	NPAR	CMIN	DF	P	CMIN/DF
Default model	46	419.225	144	.000	2.911
Saturated model	190	.000	0		
Independence model	19	4842.502	171	.000	28.319

RMR, GFI

Model	RMR	GFI	AGFI	PGFI
Default model	45.329	.892	.857	.676
Saturated model	.000	1.000		
Independence model	331.842	.209	.121	.188

Baseline Comparisons

Model	NFI Delta1	RFI rho1	IFI Delta2	TLI rho2	CFI
Default model	.913	.897	.941	.930	.941
Saturated model	1.000		1.000		1.000
Independence model	.000	.000	.000	.000	.000

Parsimony-Adjusted Measures

Model	PRATIO	PNFI	PCFI
Default model	.842	.769	.792
Saturated model	.000	.000	.000
Independence model	1.000	.000	.000

NCP

Model	NCP	LO 90	HI 90
Default model	275.225	217.703	340.386
Saturated model	.000	.000	.000
Independence model	4671.502	4448.136	4902.113

FMIN

Model	FMIN	F0	LO 90	HI 90
Default model	1.106	.726	.574	.898
Saturated model	.000	.000	.000	.000
Independence model	12.777	12.326	11.737	12.934

RMSEA

Model	RMSEA	LO 90	HI 90	PCLOSE
Default model	.071	.063	.079	.000
Independence model	.268	.262	.275	.000

AIC

Model	AIC	BCC	BIC	CAIC
Default model	511.225	516.350	692.473	738.473
Saturated model	380.000	401.170	1128.633	1318.633
Independence model	4880.502	4882.619	4955.366	4974.366

ECVI

Model	ECVI	LO 90	HI 90	MECVI
Default model	1.349	1.197	1.521	1.362
Saturated model	1.003	1.003	1.003	1.058
Independence model	12.877	12.288	13.486	12.883

HOELTER

Model	HOELTER .05	HOELTER .01
Default model	157	169
Independence model	16	17

D. Mediation Analysis Model 4 (using the Hayes SPSS Process Macro version 4.0)

```
Run MATRIX procedure:

***************** PROCESS Procedure for SPSS Version 4.0 *****************

          Written by Andrew F. Hayes, Ph.D.       www.afhayes.com
    Documentation available in Hayes (2022). www.guilford.com/p/hayes3

**************************************************************************
Model  : 4
    Y  : AIS
    X  : CBI
    M  : BRS

Sample
Size:  380

**************************************************************************
OUTCOME VARIABLE:
 BRS

Model Summary
          R       R-sq        MSE          F        df1        df2          p
      .4571      .2090      .5467    99.8567     1.0000   378.0000      .0000

Model
             coeff         se          t          p       LLCI       ULCI
constant    4.4117      .1050    42.0014      .0000     4.2051     4.6182
CBI         -.0201      .0020    -9.9928      .0000     -.0240     -.0161

Standardized coefficients
         coeff
CBI     -.4571

**************************************************************************
OUTCOME VARIABLE:
 AIS

Model Summary
          R       R-sq        MSE          F        df1        df2          p
      .6120      .3746    14.4950   112.8866     2.0000   377.0000      .0000

Model
             coeff         se          t          p       LLCI       ULCI
constant    4.8435     1.2875     3.7621      .0002     2.3120     7.3750
CBI          .1264      .0116    10.8629      .0000      .1035      .1493
BRS        -1.1301      .2648    -4.2673      .0000    -1.6509     -.6094

Standardized coefficients
         coeff
CBI      .4975
BRS     -.1954

************************** TOTAL EFFECT MODEL ****************************
OUTCOME VARIABLE:
 AIS

Model Summary
          R       R-sq        MSE          F        df1        df2          p
      .5868      .3443    15.1549   198.5244     1.0000   378.0000      .0000

Model
             coeff         se          t          p       LLCI       ULCI
constant    -.1423      .5530     -.2573      .7971    -1.2296      .9451
CBI          .1491      .0106    14.0899      .0000      .1283      .1699

Standardized coefficients
         coeff
CBI      .5868
```

************** TOTAL, DIRECT, AND INDIRECT EFFECTS OF X ON Y **************

Total effect of X on Y

Effect	se	t	p	LLCI	ULCI	c_cs
.1491	.0106	14.0899	.0000	.1283	.1699	.5868

Direct effect of X on Y

Effect	se	t	p	LLCI	ULCI	c'_cs
.1264	.0116	10.8629	.0000	.1035	.1493	.4975

Indirect effect(s) of X on Y:

	Effect	BootSE	BootLLCI	BootULCI
BRS	.0227	.0064	.0113	.0362

Completely standardized indirect effect(s) of X on Y:

	Effect	BootSE	BootLLCI	BootULCI
BRS	.0893	.0250	.0446	.1408

*********************** ANALYSIS NOTES AND ERRORS ************************

Level of confidence for all confidence intervals in output:
95.0000

Number of bootstrap samples for percentile bootstrap confidence intervals:
5000

------ END MATRIX -----
